# Toxicity of Amorphigenin from the Seeds of *Amorpha fruticosa* against the Larvae of *Culex pipiens pallens* (Diptera: Culicidae)

**DOI:** 10.3390/molecules20023238

**Published:** 2015-02-16

**Authors:** Yaping Liang, Xiuwei Li, Zumin Gu, Peiwen Qin, Mingshan Ji

**Affiliations:** 1 Department of Pesticide Science, College of Plant Protection, Shenyang Agricultural University, Shenyang 110866, China; E-Mails: lypnancy628@163.com (Y.L.); guzumin1212@163.com (Z.G.); qinpeiwen08@sina.com (P.Q.); 2 Biopesticide Engineering Research Center of Liaoning Province, Shenyang 110866, China

**Keywords:** *Amorpha fruticosa*, larvicidal activity, *Culex pipiens pallens*, amorphigenin, mitochondrial complex I, protein synthesis

## Abstract

The larvicidal activity of the crude petroleum ether, ethyl acetate, acetone, chloroform and ethanol extracts of *Amorpha fruticosa* seeds was individually assayed for toxicity against the early fourth-instar larva of the mosquito, *Culex pipiens pallens* after 24 h exposure. Of the tested extracts, the ethanol one exhibited the highest larvicidal activity (LC_50_ = 22.69 mg/L). Amorphigenin (8'-hydroxyrotenone), a rotenoid compound which exhibits a strong larvicidal activity with LC_50_ and LC_90_ values of 4.29 and 11.27 mg/L, respectively, was isolated from the ethanol extract by column chromatograpy. Its structure was elucidated by ^1^H-NMR, UV and IR spectral data. Furthermore, investigation of amorphigenin’s effects on mitochondrial complex I activity and protein synthesis in *C. pipiens pallens* larvae reveals that amorphigenin decreases mitochondrial complex I activities to 65.73% at 10.45 μmol/L, compared to the control, when NADH were used as the substrate. Meanwhile, amorphigenin at 10.45 μmol/L also caused a 1.98-fold decrease in protein content, compared to the control larvae treated with acetone only.

## 1. Introduction

Mosquitoes are significant public health pests due to their predominance as agents of potentially deadly pathogens in human beings and the annoyance of skin reactions caused by their bites [1]. *Culex pipiens pallens* Coquillett 1998, is the most common domestic mosquito species in Northern China, Korea and Japan, and is the primary vector of wuchereriasis, epidemic encephalitis B [2] and a potential vector of West Nile virus [3]. Since the 1950s, mosquito control strategies have depended primarily on the use of synthetic chemical insecticides [4,5]. However, repeated and injudicious application of synthetic insecticides has often resulted in the development of resistance to organochlorine, organophosphate, carbamate and pyrethroid insecticides [6,7,8,9]. Hence, in mosquito control, there is a constant need for developing alternatives to chemical insecticides. Natural, bio-active botanical compounds are a promising alternative for mosquito control because of lower toxicity to non-target organisms and their innate biodegradation ability [10,11].

In recent years, many studies on plant-borne compounds (e.g., essential oils and plant extracts) against a wide range of mosquito larvae have been conducted around the world [12]. Some novel mosquito larvicidal compounds were isolated and identified, and their modes of action have been investigated [13,14,15,16,17]. Among botanicals tested against mosquito larvae of *Aedes aegypti*, *Aedes togoi* and *C. pipiens pallens* are the following: methanol extracts from *Cinnamomum cassia* bark, *Illicium verum* fruit, *Piper nigrum* fruit, *Zanthoxylum piperitum* fruit, and *Kaempferia galangal* rhizome [18]; isobutylamides in *Piper nigrum* fruits [19]; active constituent isolate from *Tabebuia avellanedae* bark [20] and lignans in *Phryma leptostachya var. asiatica* root [21]. Compounds that subsequently demonstrated toxicity against larvae of *Aedes aegypti* and *C. pipiens pallens* include methanol extracts from *Kigelia pinnata* and *Ruta chalepensis* [22]; imperatorin and osthole from *Cnidium monnieri* fruit [23]; extracts from *Cassia obtusifolia*, *Cassia tora* and *Vicia tetrasperma* [24] and the chloroform fraction of *Cassia obtusifolia* extract and the biologically active component emodin [25].

The essential oil extracted from *Coriandrum sativum* fruits [26], *Hyptis suaveolens* [27], plus wild and cultivated *Ruta chalepensis* [28] were evaluated for larvicidal and repellent activities against the most invasive mosquito worldwide, *Aedes albopictus.* As well, *Salvia dorisiana*, *S. longifolia* and *S. sclarea* essential oils [29] were studied for repellent activity against *A. albopictus.* Neem seed oil [30], neem cake [31] of *Azadirachta indica* and their fractions were evaluated for larvicidal toxicity and field oviposition deterrence against *A. albopictus.* Furhtermore, essential oils extracted from *Zingiber officinalis* rhizome and leaf, plus the stem of *Achyranthes aspera* were evaluated for larvicidal, attractant/repellent, and oviposition attractant/deterrent activity against two mosquito species, *Aedes aegypti* and *Culex quinquefasciatus* [32]. The bioactivity of 14 essential oils from *Cinnamomum osmophleum*, *Taiwania cryptomerioides*, *Cunninghamia lanceolata*, *Cryptomeria japonica* and *Calocedrus formosana* were tested using the *Aedes aegypti* larvicidal assay [33]. Larvicidal activity of five medicinal plants, *Abutilon indicum*, *Aegle marmelos*, *Euphorbia thymifolia*, *Jatropha gossypifolia* and *Solanum torvum* were assayed for their toxicity against *Culex quinquefasciatus* [34]. The active compound (*Z*)-ligustilide of the chloroform extract from *Angelica sinensis* roots deterred the biting of two mosquito species, *Aedes aegypti* and *Anopheles stephensis,* more effectively than DEET [35].

As for insecticidal activity against mosquito larvae of *C. pipiens pallens*, the essential oils extracted from *Amyris balsamifera*, *Daucus carota*, and *Pogostemon cablin* were investigated [36]; Also evaluated were the crude methanol extract of *Chamaecyparis obtusa* leaves, its active component beta-thujaplicin [37] and four sesquiterpene alkaloids from an ethanol extract of *Tripterygium wilfordii* (Celastraceae) root bark [38]. Whole-plant crude petroleum ether, ethyl acetate, and methanol extracts of *Phryma leptostachya* have been tested for larvicidal activity against the early fourth-instar larvae of *C. pipiens pallens*, from which three lignans, phrymarolin-I, haedoxane A, and haedoxane E, were isolated and identified as new mosquito larvicidal compounds [39]. Extracts from seven species of bamboo were evaluated for larvicidal activity against *C. pipiens pallens*. Among these seven, extracts from *Pleioblastus juxianensis, Brachystachyum albostriatum, Phyllostachys platyglossa* and *Pleioblastus amarus* were found to be effective, with LC_50_ values at 24 h of 30.65 mg/L, 53.94 mg/L, 41.21 mg/L and 54.49 mg/L, respectively [40]. A methanol extract of *Piper longum* fruit was found to be active against mosquito larvae of *C. pipiens pallens* at 10 mg/mL after 24 h, in which the piperidine alkaloid, pipernonaline, is the active principle, with an LC_50_ of 0.21 mg/L [41]. Ethanolic extracts from the *Ginkgo biloba* L. exocarp [42], ethyl cinnamate and ethyl *p*-methoxy-cinnamate from *Kaempferia galangal* rhizome [43], plus (‒)-asarinin, α-asarone, methyleugenol, pellitorine, and pentadecane from *Asarum heterotropoides* root [44] were all assayed against larvae of *C. pipiens pallens*. Using a direct-contact mortality bioassay, the toxicity of pellitorine alone, or in combination with (‒)-asarinin, α-asarone, and methyleugenol, or pentadecane was evaluated on third instars from an insecticide-susceptible KS-CP strain and a resistant DJ-CP colony of *C. pipiens pallens* [45].

*Amorpha fruticosa* is a perennial deciduous shrub in the Leguminosae family [46], introduced into China around the 1920s and widely planted throughout China for erosion control and to restore wasteland. Its fresh fruits were used for the treatment of carbuncles, eczema and burns [47]. Earlier research in the 1940s showed that extracts of *A. fruticosa* possess repellent and insecticidal activity against various insect species [48,49]. The acetone extract of *A. fruticosa* seeds was proved to be more toxic against *A. aegypti* larvae than 1% pure rotenone [49]. In our recent research [50], we found that ethanol extract from the seeds of *A. fruticosa* have good contact effects and anti-feedent activity against *Schizaphis graminum.* In the present study, our main target concentrated on larvicidal activity assessment of extracts and the isolated compound amorphigenin from the seeds of *Amorpha fruticosa* against early fourth-instar larvae of *C. pipiens pallens*. Moreover, expected effects of amorphigenin on mitochondrial complex I activity and protein synthesis of *C. pipiens pallens* were investigated to confirm its mode of action. Results from this study are expected to provide insights into mosquito larvicidal activity of seeds of *Amorpha fruticosa* and potential inhibition of amorphigenin against mitochondrial complex I and protein synthesis of *C. pipiens pallens*.

## 2. Results and Discussion

### 2.1. Structural Elucidation of Amorphigenin

**Figure 1:**
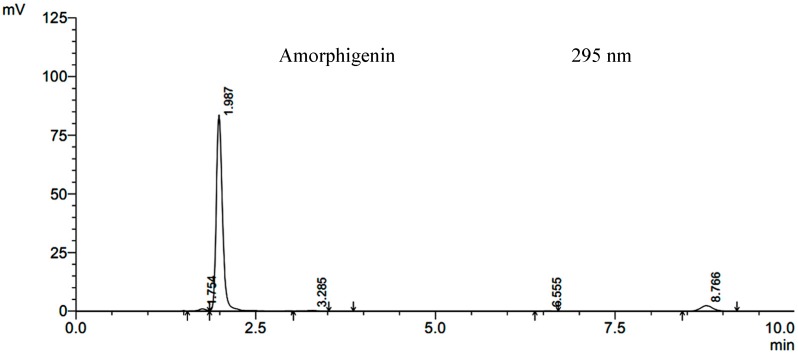
HPLC chromatogram of amorphigenin.

**Figure 2:**
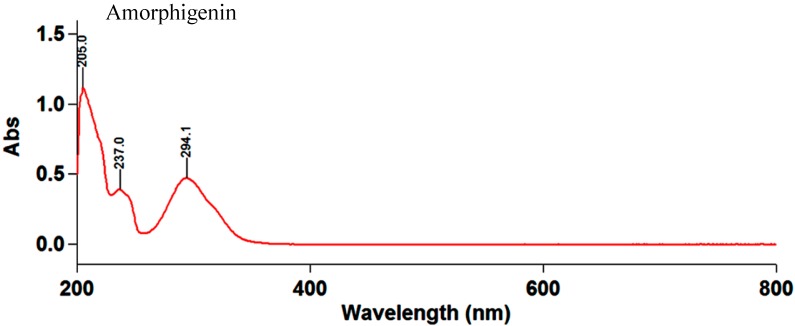
UV spectrum of amorphigenin.

**Figure 3:**
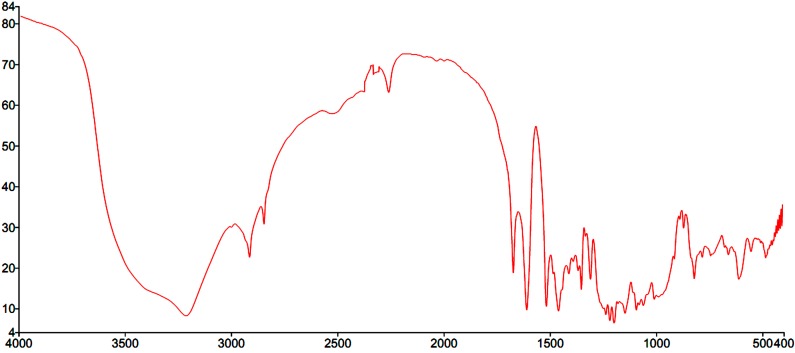
IR spectrum of amorphigenin.

**Figure 4:**
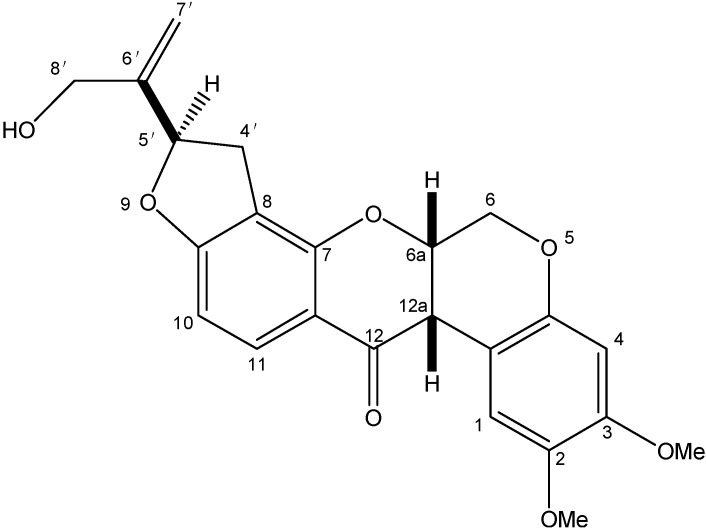
Chemical structure of amorphigenin.

The sample obtained from preparative high performance liquid chromatography was recrystallized from hot methanol to yield a sample of creamy white needles, which showed a single spot on thin-layer chromatography (TLC) over silica gel (*Rf* = 0.45) with benzene/methanol (9:1) as the developing system, and a single HPLC peak (Figure 1). This compound possesses following physical properties: a melting point of 183 °C, UV (MeOH) λmax = 205, 237 and 294.1 nm (Figure 2) and an obvious OH-stretching band at 3500 to 3400 cm^−1^ in its IR spectrum (KBr disk, Figure 3), while other bands were similar to those of rotenone. ^1^H-NMR (δ, CDCl_3_, 600 MHz) = 6.75 (1H, s, H-1), 6.46 (1H, s, H-4), 4.19 (1H each, dd, *J* = 12.6 and 2.4, H-6) 4.63 (1H each, dd, *J* = 12.6 and 3, H-6), 4.94 (1H, m, H-6a), 6.52 (1H, d, *J* = 8.4, H-10), 7.85 (1H, d, *J* = 8.4, H-11), 3.09 (1H each, dd, *J* = 15.6 and 8.4, H-4'), 3.45 (1H each, dd, *J* = 15.6 and 9.6, H-4'), 5.44 (1H, t, *J* = 7.8, H-5'), 5.29 (1H, s (br), H-7'), 4.27 (1H, s (br), H-8'), 3.76 and 3.85 (both 3H, s, OMe), 1.43 (1H, s(br), 8'-OH), see Supplementary Material (Figure S1 ^1^H-NMR spectrum of amorphigenin). According to the similarity of spectral data to that reported by Sariaslani *et al*. [51] and Abe *et al*. [52], the abovementioned data, suggest that the obtained compound was identical to the known rotenoid, amorphigenin (Figure 4).

Previous research showed that amorphigenin 8'-β-glucoside can cause 85% mortality of the 4th instar larvae of *A. aegypti* and termination of pupal formation at 10 mg/L [52], in the current research, the IR spectra and ^1^H-NMR analysis of the bioactive compound indicated that a rotenoid compound, amorphigenin, is one of the main secondary metabolites responsible for larval mortality. Amorphigenin (also known as 8'-hydroxyrotenone), an aglycone of the rotenoid glycoside amorphin [53,54], was isolated from the leaves, seeds and seedlings of *A. fruticosa* [55,56] and shown to have significant anti-proliferative activity [57], anti-cancer activity in many cell types [58,59], hepatoprotective activity [60] and neuraminidase inhibition activity [61]. In our research, the mosquito larvicidal activity of amorphigenin was confirmed, and the LC_50_ value of 4.29 mg/L was recorded for early fourth-instar larvae of *C. pipiens pallens* after 24 h of exposure, which indicate its highest effect in low dosage.

### 2.2. Effect of Extracts and Amorphigenin on Larval Mortality

In the preliminary screening of botanical extracts, all extracts from the seeds of *A. fruticosa* showed moderate larvicidal activities against *C. pipiens pallens* in 24 h at 150 mg/L (Table 1), of which the ethanol extract had the most pronounced larvicidal activity, with 94.21% mortality. The corresponding LC_50_ values of the petroleum ether, ethyl acetate, chloroform, acetone, and ethanol extracts were 33.02, 86.16, 36.43, 34.23, and 22.69 mg/L, respectively (Table 2). In other words, the descending order of toxicity is ethanol extract followed by petroleum ether extract, acetone extract, chloroform extract and ethyl acetate extract, the respective, relative potency of these being 1.00, 1.46, 1.51, 1.61 and 3.80. Further analysis of relative median potency (Table 3) indicates that the LC_50_ values show significant differences for the different extracts.

**Table 1:** Preliminary screening of different solvent extracts of *Amorpha fruticosa* L. against early fourth instar larvae of *Culex pipiens pallens* at 150 mg/L.

Material	Solvents	% Mortality ^a^(mg/L) ± SE
Seeds of *Amorpha fruticosa*	Petroleum ether	92.33 ± 0.1949
	Ethyl acetate	69.77 ± 1.0632
	Chloroform	87.89 ± 0.9112
	Acetone	91.26 ± 0.3515
	Ethanol	94.21 ± 0.2631

Note: ^a^ Mean value of three replicates.

**Table 2:** LC_50_ and LC_90_ values for the larvicidal activity of extracts and amorphigenin compound from the seeds of *A. fruticosa* against early fourth instar larvae of *C. pipiens pallens*.

Treatment	LC_50_ (mg/L)	LCL-UCL ^a^ (mg/L)	LC_90_ (mg/L)	LCL-UCL (mg/L)	Regression Equation	R value
Petroleum ether	33.02	19.73–55.27	128.45	76.74–215.01	Y = 1.7008 + 2.1723X	0.995
Ethyl acetate	86.16	49.78–149.15	339.78	196.30–588.15	Y = 0.8375 + 2.1508X	0.995
Chloroform	36.43	25.68–51.67	171.78	121.11–243.64	Y = 2.0287 + 1.9029X	0.999
Acetone	34.23	17.18–68.20	138.19	69.35–275.38	Y = 1.7558 + 2.1144X	0.992
Ethanol	22.69	11.45–44.96	105.78	53.39–209.58	Y = 2.4009 + 1.9169X	0.979
Amorphigenin	4.29	3.22–5.72	11.27	8.46–15.01	Y = 3.0665 + 3.0576X	0.993
Rotenone	4.69	3.58–6.15	12.20	9.31–15.99	Y = 2.9259 + 3.0887X	0.999

Note: ^a^ LCL represents the lower confidence limit, UCL represents the upper confidence limit based on a 95% Confidence interval.

**Table 3:** Relative median potency analysis comparing toxicity of different extracts from the seeds of *A. fruticosa* against early fourth instar larvae of *C. pipiens pallens*.

Treatment ^a^	PE	EAC	MT	DMK
EAC	0.681 ^b^	-	-	-
MT	1.018	1.538	-	-
DMK	1.050	1.587	1.032	-
EA	1.206	1.949	1.267	1.228

Notes: PE = Petroleum ether extract from the seeds of *A. fruticosa*; EAC = Ethyl acetate extract from the seeds of *A. fruticosa*; MT = Chloroform. ether extract from the seeds of *A. fruticosa*; DMK = Acetone extract from the seeds of *A. fruticosa*; EA = Ethanol extract from the seeds of *A. fruticosa.* ^a^ Comparison between treatment (row *vs*. column) probit analyses of larvicidal activity. ^b^ Values < 1 indicate that extract in row is more toxic than extract in column.

Exposure of *C. pipiens pallens* larvae to amorphigenin increased mortality in a concentration-dependent manner. After 24 h of larval exposure, the LC_50_ and LC_90_ values were 4.29 and 11.27 mg/L, respectively (Table 2). Comparing these results to rotenone, which recorded 4.69 and 12.20 mg/L for LC_50_ and LC_90_ values, respectively, against early fourth-instar larvae of *C. pipiens pallens*. However, the statistical analysis showed that significant level value is greater than 0.05 (sig = 0.064), there is no significant difference between amorphigenin and rotenone. The results support the view that the plant *A. fruticosa* harbors potentially bioactive substances against mosquito larvae.

### 2.3. Effect of Amorphigenin in Vivo on Inhibition of Mitochondrial Complex I

**Figure 5:**
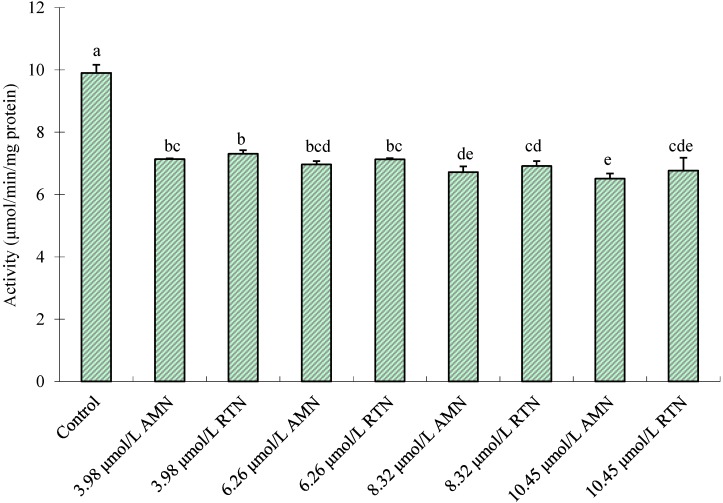
*In vivo* inhibition of mitochondrial complex I extracted from fourth-instar *C. pipiens pallens* by three different concentrations of amorphigenin (AMN) and rotenone (RTN) using NADH as substrates. Vertical bars indicate standard errors of the mean (*n* = 3). Different letters on the bars indicate that the means are signiﬁcant different among the treatments in Fisher’s LSD multiple comparison tests (*p* < 0.05).

Results showed that amorphigenin at 6.26, 8.32 and 10.45 μmol/L decreases mitochondrial complex I activities to 70.33%, 67.88% and 65.73%, respectively, comparable with the control when NADH were used as the substrate (Figure 5). Similarly, rotenone decreases mitochondrial complex I activities to 72.00%, 69.85% and 68.34%, respectively. Mitochondrial complex I (NADH-ubiquinone oxidoreductase, EC 1.6.5.3) catalyzes the transfer of two electrons from NADH to ubiquinone [62]. Inhibition of complex I results in the termination of ATP production. It is believed that rotenone and rotenoids are acting between NADH and coenzyme Q [63]. An *in vitro* study by Earley *et al.*, showed that amorphigenin and its analog, arylazidoamorphigenin, are potent inhibitors of mitochondrial complex I, comparable to rotenone [64]. With a degree of inhibition similar to that of rotenone, our study shows that amorphigenin is a potent inhibitor against mitochondrial complex I of *C. pipiens pallens* larvae. The inhibition similarities of mitochondrial complex I can be attributed to similar chemical structures of amorphigenin and rotenone. Spectroscopy research indicates that amorphigenin differs from rotenone in the presence of a hydroxy group in the substituent locus on the E ring [56]. The results suggest that amorphigenin may present the same mode of action as rotenone. 

### 2.4. Effect of Amorphigenin on Protein Production

**Figure 6:**
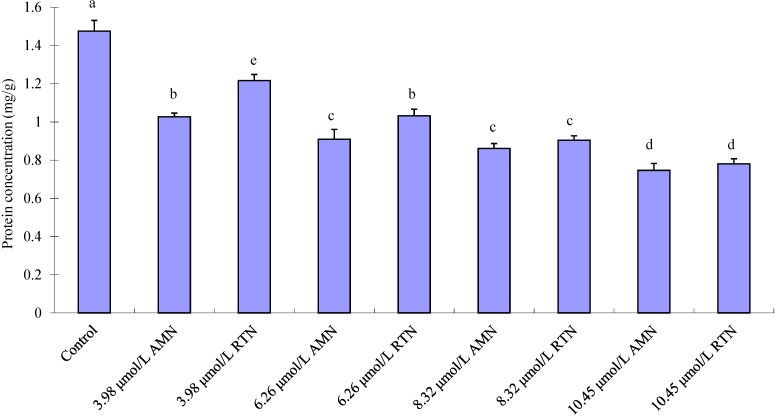
Comparison of total protein concentrations among the control, Amorphigenin (AMN) and Rotenone (RTN) treated fourth-instar larvae of *C. pipiens pallens* following 24-h exposure. Vertical bars indicate standard errors of the mean (*n* = 3). Different letters on the bars indicate that the means are signiﬁcant different among the treatments in Fisher’s LSD multiple comparison tests (*p* < 0.05).

Amorphigenin at 6.26, 8.32 and 10.45 μmol/L respectively decreased the protein content by 1.62-, 1.71-, and 1.98-fold, in comparison with control rates (Figure 6). Rai and Carpinella [65] mentioned that rotenone inhibits the assembly of spindle body canaliculi and the formation of canaliculi of insects *in vitro* by reversible conjugation with canaliculus protein, changes the components of protein in the integument of Lepidoptera larvae, and decreases the whole protein content. In this research, amorphigenin and rotenone at 10.45 μmol/L decreased the protein content of early fourth-instar larvae of *C. pipiens pallens* by 1.98- and 1.89-fold, respectively. In consideration of similarity between chemical structures of amorphigenin and rotenone, amorphigenin may inhibit protein synthesis in the integument and decrease the overall protein content of mosquitoes. 

### 2.5. Discussion

Because they demonstrate a wide range of bioactivity and possess contact and fumigant toxicity, plus repellent, oviposition, and feeding deterrence against various mosquito species, several botanicals have been considered as potential mosquito larvae control agents. The use of the plants to control mosquito larvae has been investigated frequently and is well documented by Kishore *et al*. [66]. Plant secondary metabolites have been increasingly used, given their beneﬁt in mosquito larva control, however, these substances constitute a substantial source of contamination to non-target aquatic organisms that share the same ecological niche as mosquitoes. *Moringa oleifera* has been used as a coagulation reagent for drinking water puriﬁcation, and can be used as bio-insecticidal tool with potential for the control of insects [67]. However, signiﬁcant cytotoxic effects were observed from powdered *M. oleifera* seeds at concentrations from 1 to 50 mg/L [68]. Research by Conti *et al*. [69] demonstrates that *Melaleuca alternifolia* essential oil has a remarkable acute toxicity toward the non-target water flea, *Daphnia magna*, with an LC_50_ of 80.636 mg/L. The water flea shares the same ecological niche as *A. albopictus* larvae. Thus, non-target effects of natural products against aquatic organisms must be considered in the development of eco-friendly mosquito control strategies. Further research is needed to investigate toxicity of plant secondary metabolite both on target and non-target aquatic arthropods.

## 3. Experimental Section

### 3.1. Plant Materials

In October 2013, the dried seeds of *A. fruticosa* were collected from the campus of Shenyang Agricultural University in Liaoning Province, China (41°49'50.10''N, 123°33'53.97''E, 41.6 m altitude), air-dried at room temperature, then ground with an airflow grinder (YF111b, Yongli, Ruian, China). The seeds were authenticated by Associate Professor Guanghui Tang of the College of Forestry, Northwest A&F University. Voucher specimens have been deposited at The Bio-Pesticide Engineering Research Center of Liaoning Province, Shenyang Agricultural University. 

### 3.2. Mosquito Culture

*C. pipiens pallens* were maintained in our laboratory without exposure to any insecticide at 27 ± 2 °C with 75%–85% relative humidity and a 12:12 L/D photoperiod. The larvae of *C. pipiens pallens* were reared in a plastic basin containing a sterilized diet (40 mesh goat liver powder/yeast (2:1) in water). Adult mosquitoes were maintained on a 10% sucrose solution and blood from a live mouse.

All procedures performed on animals within this study were conducted following guidelines of the Association for Assessment and Accreditation of Laboratory Animal Care International (AAALAC).

### 3.3. Preparation of Plant Extracts

The dried seeds of *A. fruticosa* were finely powdered. Using an ultrasonic processor (KQ-500DE, Kunshan, China), 500 g of powdered seed was thrice extracted in sequence (60 min per run) by 1500 mL of petroleum ether organic solvents (60–90 °C), ethyl acetate, chloroform, acetone, and ethanol. The ultrasonic extraction power was set at 350 W and a frequency of 40 KHz. The extract was filtered and concentrated using a rotary vacuum evaporator, and the residue obtained was stored at 4 °C. During preliminary screening, the ethanol extract exhibited the highest larvicidal activity (LC_50_ = 22.69 mg/L) against the early 4th-instar larvae of *C. pipiens pallens*. Thus the ethanol extract was selected for further isolation and purification by column chromatography and preparative high performance liquid chromatography methods.

### 3.4. Isolation and Purification of Active Ingredients

The ground seeds of *A. fruticosa* (1 kg) were extracted three times (three days each time) by ethanol at room temperature. Evaporation of the ethanol yielded a residue (144.88 g), from which a portion (50 g) was chromatographed on a silica gel column (9.0 × 150 cm) using chloroform/methanol (20:1–3:1, v/v) as eluant. After analysis by thin-layer chromatography, fractions with similar Rf values were combined to provide seven fractions (I–VII). The larvicidal activities of the fractions I–VII against fourth-instar larvae of *C. pipiens pallens* were tested, wherein fraction I demonstrated strong activity. Next, fraction I (6.87 g) was subjected to serial column chromatography, with varying proportions of benzene and acetone as eluants, to isolate the bioactive fraction, which was then purified by preparative high-performance liquid chromatography. For this process, a Globalsil ODS C18 column was used (10 × 250 mm, 5 μm), with methanol/water (7:3, by volume), at a flow rate of 3 mL/min, and detection at 295 nm. Finally, the potent active rotenoid, amorphigenin was isolated.

The structure of amorphigenin was elucidated mainly by analysis of its NMR spectral data. The melting point was measured on an X-5 melting-point apparatus (Yuhua, Shanghai, China) and is uncorrected. The UV spectrum was obtained on a Cary-50 spectrophotometer (Varian, Palo Alto, CA, USA). The IR spectrum was determined using a PerkinElmer spectrum65 spectrophotometer (Foster City, CA, USA) with potassium bromide pellet. The target compound was isolated and purified on a Shimadzu 2010A HT HPLC apparatus (Kyoto, Japan), equipped with a C18 preparative column (10 × 250 mm, 5 μm), with MeOH/H_2_O = 7:3 as eluant and UV detection at 295 nm. The ^1^H-NMR spectrum was recorded on a Bruker-Avance-600 spectrometer (Fällanden, Switzerland), with CDCl_3_ as solvent and tetramethylsilane (TMS) as internal standard.

### 3.5. Larvicidal Bioassay

The larvicidal activity test was conducted according to the World Health Organization standard protocols [70], with slight modifications. The compound amorphigenin was extracted from seeds of *A. fruticosa* and rotenone was purchased from Guangxi Shile Agrochemical Co., Ltd (Nanning, China). Briefly, each extract, amorphigenin and rotenone were serially diluted from a stock solution prepared in alcohol. Then, 1 mL of the serially-diluted extract and compounds were transferred into 200 mL of distilled water in a 250 mL sterile glass beaker. The concentrations of the extracts in beaker ranged from 1 mg/L to 10 mg/L, while the compounds amorphigenin and rotenone in beaker ranged from 10 mg/L to 100 mg/L. Twenty early fourth-instar larvae of *C. pipiens pallens* were separately introduced into different glass beakers. A separate set of beakers received alcohol only and served as controls. Treated and control larvae were kept under the same conditions used for mosquito maintenance. Mortality rate was recorded at 24 h after treatment. Dead larvae were identified by failure to move when probed with a needle. The experiments were replicated three times. 

### 3.6. Assay of Mitochondrial Complex I Activity

The fourth-instar larvae of *C. pipiens pallens* were exposed to sub-lethal concentrations of amorphigenin and rotenone for 24 h. The sub-lethal concentrations [71,72] (LC_10_, LC_25_, LC_40_, LC_50_ at 1.63, 2.57, 3.41, 4.29 mg/L, respectively) of amorphigenin were 3.98, 6.26, 8.32 and 10.45 μmol/L. The same mole concentration was employed for rotenone. Next, live larvae were collected and isolation of the mitochondria was performed according to Akbar *et al.* [73]. The Protein concentration of mitochondria was determined by Smith’s method [74], using bovine serum albumin (BSA) as standard. Mitochondrial complex I activity of *C. pipiens pallens* was determined as described by Birch-Machin *et al.* [75] with adaptations for a 96-well format. In brief, 20 μL mitochondria and 160 μL assay buffer, containing 25 mM phosphate buffer (pH 7.2), 5 mM MgCl_2_, 2 mM NaN_3_, 2.5 mg/mL BSA, were added to each well of a 96-well plate, then the microplate was incubated for 20 min at 37 °C in a microplate reader (Spectramax 190 plate reader, Molecular Devices, Sunnyvale, CA, USA). Finally, the reaction was initiated by the addition of 10 μL of 0.13 mM NADH and measured for 5 min.

### 3.7. Protein Assay

Surviving larvae, collected in bioassay of mitochondrial complex I activity, were homogenized in 0.1 M phosphate buffer (pH 7.0) containing 0.5% (v/v) Triton X-100 at the rate of 100 μL per larva. After the homogenates were centrifuged at 15,000× *g* for 15 min at 4 °C, the supernatants were transferred to new tubes for protein determination [76]. The concentration of total protein in each sample preparation was determined based on the method of Smith *et al*. [74] using bovine serum albumin as standard. Using the microplate reader mentioned above at 562 nm, measurement was performed. 

### 3.8. Statistical Analysis 

Applying Microsoft Excel 2003 software, the average larval mortality results, adjusted by Abbott [77], were subjected to probit analysis to calculate LC_50_ and LC_90_ with their lower and upper confidence limits based on 95% confidence intervals and correlation coefficient. Significant differences between LD_50_ values were determined by estimation of confidence intervals of the relative median potency. Two-way analysis of variance (ANOVA) and Fisher’s least signiﬁcant difference (LSD) multiple comparisons were then used to separate the means among the treatments by using the PASW Statistics 18.0 software.

## 4. Conclusions 

A considerable number of plant secondary metabolites have proven to be safe and effective against mosquito larvae. However, alternative means of control are being sought due to the dramatic increase in resistance of mosquitoes to chemical insecticides. The present study represents a systematic isolation and identification of bioactive compounds having mosquito larvicidal activity from the seeds of *A. fruticosa*. This study improves knowledge of insecticidal constituents extracted from the seeds of *A. fruticosa*, and the potent effect of amorphigenin on mitochondrial complex I activity and protein content of *C. pipiens pallens* larvae. 

Investigation of larvicidal activity against *C. pipiens pallens* demonstrates that *A. fruticosa* extract has potent insecticidal properties, confirming previous evidence. The insecticidal compound, amorphigenin, exhibits obvious larvicidal activity against *C. pipiens pallens*, and decreases mitochondrial complex I activity as well as protein content of these pests. Our results indicate that amorphigenin is strong candidate for a natural, safe and effective phyto-larvicide to be used in population control of *C. pipiens pallens.*

## Supplementary Materials

Supplementary materials can be accessed at: http://www.mdpi.com/1420-3049/20/02/3238/s1.
