# Supplementary figures and images for: Toxicity of Amorphigenin from the Seeds of Amorpha fruticosa against the Larvae of Culex pipiens pallens (Diptera: Culicidae)
Source: Molecules. 2015 Feb 16;20(2):3238–54. doi: 10.3390/molecules20023238 (PMC6272459; doi:10.3390/molecules20023238)

# Supplementary Materials

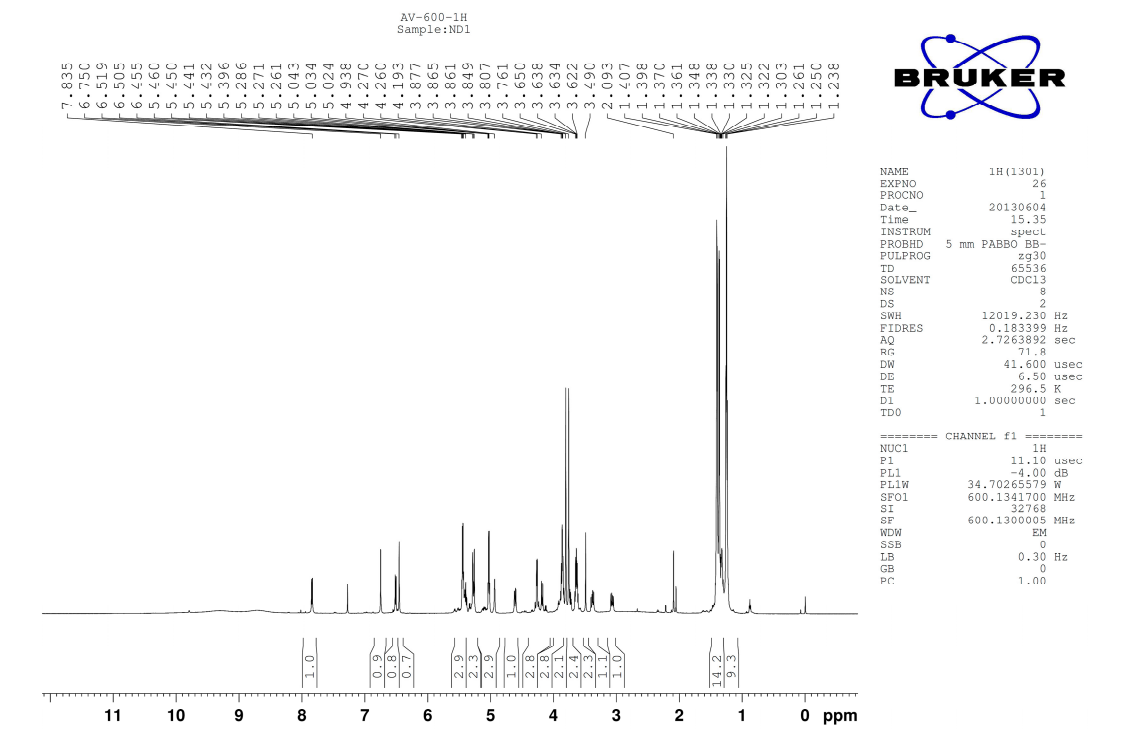

Figure S1. <sup>1</sup>H-NMR spectrum of amorphigenin.

Supplement: Supplementary file 1 [file molecules-20-03238-s001.pdf]
